# The Intergenerational Impact of Maternal Childhood Adversity on Child Behavior and Neurodevelopment: The Healthy MiNDS Protocol

**DOI:** 10.1002/mpr.70031

**Published:** 2025-07-19

**Authors:** Danilo Micali, Ana Carolina Coelho Milani, Camilla Salmeron, Célia Araújo, Aline Camargo Ramos, Marcos Roberto Fanton, Sara B. VanBronkhorst, Nitamar Abdala, Ivaldo Silva, Sintia Iole Belangero, Cristiane S. Duarte, Jonathan Posner, Andrea Parolin Jackowski

**Affiliations:** ^1^ Laboratory of Integrative Neuroscience (LiNC) Universidade Federal de São Paulo São Paulo Brazil; ^2^ Department of Genetics and Morphology Universidade Federal de São Paulo São Paulo Brazil; ^3^ Department of Psychiatry Universidade Federal de São Paulo São Paulo Brazil; ^4^ Department of Pediatrics Universidade Federal de São Paulo São Paulo Brazil; ^5^ Department of Gynaecology Universidade Federal de São Paulo São Paulo Brazil; ^6^ New York State Psychiatric Institute Columbia University Irving Medical Center New York New York USA; ^7^ Department of Radiology Universidade Federal de São Paulo São Paulo Brazil; ^8^ Duke University Medical Center Durham North Carolina USA

**Keywords:** adverse childhood experiences, child behavior, fetal programming, neurodevelopment, offspring health

## Abstract

**Objectives:**

Here we present *Healthy MiNDS*, a cohort of mothers and their newborns in São Paulo, Brazil, designed to investigate how maternal adverse childhood experiences (ACEs) intergenerationally affect child behavior and neurodevelopment, while exploring underlying biological mechanisms.

**Methods:**

The study included seven encounters, beginning with the enrollment of women at 25–39 weeks of gestation from a low‐resource area, based on their high or low exposure to ACEs. Their newborns were followed through the first 2 years of life. Biospecimens (e.g. maternal and cord blood, hair, saliva, placenta) were collected before/during childbirth and at follow‐up visits. Newborns underwent non‐sedated brain MRI scans and were regularly assessed for behavior, mother‐child interactions, and home environment.

**Results:**

We enrolled 626 mothers, with 60% of those who provided ACEs information (*n* = 603) reporting three or more ACEs, and 545 mother‐child dyads. We obtained 303 MRI scans and 333 placental samples, among other biospecimens. Enrollment and the 6‐month follow‐up are complete, while the 14‐, 18‐, and 24‐month visits are ongoing for active dyads.

**Conclusion:**

The *Healthy MiNDS* data allows for testing associations between maternal ACEs, prenatal inflammation and stress, placental biology, and offspring brain‐behavior development in a population highly exposed to ACEs.

## 1 Introduction

A high percentage of children in the US (around 40%–50%) (Felitti et al. 2019; Hussey et al. 2006) have experienced one or more adverse childhood experiences (ACEs), such as physical/sexual abuse or parental mental illness. In developing countries, such as Brazil, this percentage is over 80% (Soares et al. 2016). Experiencing ACEs is significantly associated with higher risk for ADHD and other externalizing disorders [odds ratio (OR) 1.5–6.8] (Briggs‐Gowan et al. 2010), depression (OR 1.6–3.8) (Houtepen et al. 2020), low educational attainment (OR 1.7–2.4) (Houtepen et al. 2020), substance use disorders (OR 2.3–7.7) (Dube et al. 2003), risky sexual behaviors and sexually transmitted diseases (OR 1.7–8.1) (Hillis et al. 2000), and other conditions such as cardiovascular (OR 1.7–2.6) and respiratory disease (OR 2.5–3.8) (Godoy et al. 2021; Hughes et al. 2017).

Beyond the negative effects of ACEs on individuals who have been directly exposed, evidence points to intergenerational effects of maternal ACEs. For example, preclinical studies indicate that exposure to prenatal maternal stress (a possible consequence of maternal ACEs) can induce long‐term changes in various neurobiological systems of the offspring, including the hypothalamic–pituitary–adrenal (HPA) axis and related neural substrates (Bale 2015; Buss et al. 2017; Demers et al. 2022; Hendrix et al. 2022; Kim et al. 2015; Scorza et al. 2019). Similar effects are beginning to be documented in humans. For example, descendants of Holocaust survivors, compared to subjects who were not offspring of Holocaust survivors, exhibit lower cortisol levels, a trait linked to post‐traumatic stress disorder (PTSD), irrespective of their individual PTSD status (Yehuda et al. 2001; Yehuda et al. 1998). This echoes the potential transgenerational impact of traumatic events, as observed in instances such as the Vietnam War (Rosenheck and Nathan 1985) and the US Civil War (Costa et al. 2018). While investigations specifically addressing adverse childhood exposures (as opposed to those in adulthood) are scarce, those available reinforce the notion of intergenerational effects (Bouvette‐Turcot et al. 2013). However, the biological mechanisms underlying these intergenerational effects remain poorly understood in humans, with some investigations hinting at DNA methylation as a potential mediating pathway (Bowers and Yehuda 2016; Yehuda et al. 2016).

Exposure to adverse events during childhood is associated with later alterations in biological predictors of health, including inflammatory markers, cortisol, and oxytocin. These biological changes may mediate the relationship between ACEs and poor child and adult health (Danese et al. 2007; Silva et al. 2021). For example, childhood maltreatment predicts elevated levels of C‐reactive protein (CRP) 20 years later, independent of stressors in adulthood (Danese et al. 2007). Elevated adult inflammation associated with childhood trauma has been supported by meta‐analyses of CRP (18 studies, 16,870 adults), interleukin‐6 (IL‐6; 15 studies, 3751 adults) and tumor necrosis factor‐α (TNF‐α; 10 studies, 881 adults) (Baumeister et al. 2016). Similarly, meta‐analyses have found associations between early life stress and lower oxytocin levels (Ellis et al. 2021) and between childhood maltreatment and lower morning cortisol levels (Bernard et al. 2017).

Given the chronic biochemical changes associated with ACEs, fetuses of pregnant women with a history of ACEs may be exposed to a prenatal environment with alterations in inflammatory markers, cortisol, and oxytocin. This prenatal exposure may, in turn, influence fetal brain development. For instance, infants exposed to high maternal IL‐6 and CRP show atypical brain development with altered connectivity within prefrontal, temporoparietal, and insular cortices as measured by functional MRI (Rudolph et al. 2018; Spann et al. 2018). However, studies examining the intergenerational impact of ACEs remain scarce, and these studies are often limited by, for example, small samples, measuring maternal exposure to ACEs only in adulthood, and both exposure to ACE's and child outcomes based on self‐report by the mother. These limitations render the specific biological mechanisms underlying how maternal ACEs impact child development unclear.

Fetal programming is a conceptual framework that helps to understand how ACEs would be “communicated” to the offspring via maternal‐fetal transmission (O’Donnell and Meaney 2017; Seckl and Holmes 2007) impacting neurodevelopment. In this scenario, the placenta occupies a crucial role given the amount of brain development that happens in utero. By providing oxygen and nutrients—among other molecules—to the fetus during gestation, the placenta is essentially linked to fetal neurodevelopment while conferring sex‐specific responses (Burton et al. 2014; Rosenfeld 2015).

During gestation, altered levels of inflammatory markers are posited to impact placental biology and therefore fetal development. The literature on maternal ACEs, fetal programming and neurodevelopment could benefit from more studies combining (i) neuroimaging data obtained near childbirth—thus with less influence from early postnatal environmental factors—such as (Hendrix et al. 2021; Lugo‐Candelas et al. 2023) with (ii) comprehensive child behavioral measures. Although such an approach has been explored (Spann et al. 2018), still missing are human studies that link maternal inflammation to placental biology and placental biology to fetal neurodevelopment and child behavior.

With the goal of addressing this gap by investigating potential mechanisms underlying the impact of maternal ACEs on child neurodevelopment, the ‘Healthy’ MiNDS (Mother Influences on Child Neurobehavioral Development Study) cohort was established. Healthy MiNDS is a multicentered study led by researchers at Universidade Federal de São Paulo (Brazil), New York State Psychiatric Institute (USA) and Duke University School of Medicine (USA). The study specifically focuses on a sample from low‐resource settings in the cities of São Paulo and Guarulhos, Brazil, where ACEs are prevalent and prevention can have its most substantial impact. In this report, we provide details on the design of this cohort, a brief description of its current sample and discuss its potential contributions to the current literature.

## 2 Method

### 2.1 Study Overview

The study enrolled women with low‐risk pregnancies with and without a history of ACEs, from high‐risk, low‐resource settings in the cities of Guarulhos and São Paulo, in the state of São Paulo, Brazil. Participants were recruited from Brazil's unified health system (Sistema Único de Saúde, SUS) (Castro et al. 2019), a nationwide public healthcare system that offers free services to all individuals. Participants' children were enrolled at birth and followed for at least the first 2 years of life. Children's secondary caretakers were enrolled at the first home visit and then followed throughout for the remainder of the study.

**FIGURE 1:**
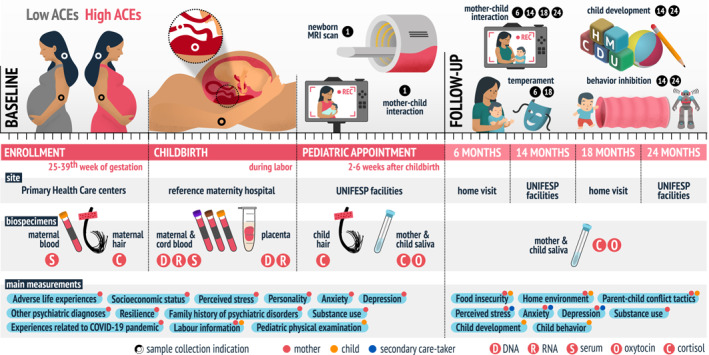
Visual overview of healthy MINDS design and procedures. Participants met our study team at enrollment during pregnancy, at childbirth, at pediatric appointments, and during follow‐up visits for the collection of biospecimens and clinical interviews. Black dots in the baseline section indicate from where biospecimens were collected. Numbered black circles indicate the number of months after childbirth at which each measurement was obtained. Follow‐up visits occurred at 6, 14, 18 and 24 months either at the participants' homes or at our research facilities. Images in the follow‐up section illustrate (part of) the protocols used to assess the indicated measurements. The main assessments related to mothers (red‐tagged), their children (orange‐tagged) and their secondary caretakers (blue‐tagged) at baseline and follow‐up visits are listed under “main measurements'' in the lower part of the figure. ACEs: adverse childhood experiences. MRI: magnetic resonance imaging. UNIFESP: Universidade Federal de São Paulo.

During baseline, our study team met participants at enrollment during pregnancy, during labor and 2–6 weeks after childbirth. Follow‐ups were then performed at 6, 14, 18 and 24 months after childbirth. Biospecimens were collected during pregnancy, labor and postnatally (Figure 1).

To ensure transparent and comprehensive reporting of this cohort, we followed the Strengthening the Reporting of Observational Studies in Epidemiology (STROBE) guidelines (STROBE Initiative 2025; von Elm et al. 2007).

### 2.2 Enrollment

We enrolled women aged 18–38 years between the 25^th^ and 39^th^ week of a low‐risk pregnancy who were able to understand the terms and conditions of the study and provide written informed consent. Pregnant women were excluded if they: (1) presented severe psychiatric condition such as schizophrenia, persistent delusional disorder, bipolar disorder, obsessive‐compulsive disorder, dementia or suicidal ideation; (2) had a history of head trauma with diagnosed brain injury, treatment for epilepsy or history of neurosurgery; (3) had decompensated clinical diseases that required intensive treatment; (4) used illicit drugs other than cannabis; (5) had a history of TORCH infections during pregnancy.

Participants were enrolled in the cities of Guarulhos and São Paulo. In Guarulhos, enrollment was conducted in four Primary Health Care (PHC) centers. PHC centers serve as important gateways to the healthcare system and eligible women were approached during their regular prenatal appointments. Pregnant women followed in these PHC centers were referred to Hospital Maternidade Jesus, José e Maria, in Guarulhos for childbirth. In São Paulo, enrollment and child births took place at Hospital Amparo Maternal. In both sites, enrollments were conducted by trained psychologists. Maternal exposure to ACEs was assessed on the enrollment day.

Newborns of participating women were enrolled at birth after confirmation of the mother's interest in continuing in the study. Mother‐child dyads were excluded if the newborn was premature (born before 37 weeks of gestation), born with low weight (below 2.5 kg), had a 5‐min Apgar below 7, was admitted to the neonatal intensive care unit, or presented with kernicterus or inborn errors of metabolism.

A secondary caretaker of the enrolled child (e.g. the other parent, a grandparent, a relative or a significant other) was enrolled at the home visit (6‐month follow‐up) after providing written consent, with a repeat assessment during the second home visit (18‐month follow‐up).

### 2.3 Enrollment Targets

We estimated our sample size based on data from a pilot study with the same design conducted in a low‐resource setting in São Paulo, Brazil (*N* = 62) in 2018. Aiming to have more than 80% power in all our analyses, our target was a cohort of 320 mother‐child dyads at the last follow‐up (24 months after childbirth). A description of power calculations for the most relevant comparisons of this study are provided in the Supplementary Material (**Item 1.2**).

To meet this aim, we initially intended to screen 720 pregnant women and enroll 580 (80% of those screened). Given the distribution of ACEs in São Paulo and Guarulhos, we expected the screening to yield around 50% in both the high (3 or more) and low (0–2) ACEs groups. After birth, we expected to obtain 400 infant MRI scans (around 70% of the 580 enrolled) yielding usable MRI data (e.g. no excessive head motion) in around 320 infants. We planned to continue to follow all 400 infants after the MRI session and estimated that we would retain 320 infants for the 24‐month follow‐up (80% of the 400 infants being followed).

The cutoff point of three or more ACEs for the high ACEs group was based on observations of a metanalysis (Hughes et al. 2017) which noted that outcomes such as mental illness, substance use, and chronic diseases increase notably with ≥ 3 ACEs. Also, the CDC's Behavioral Risk Factor Surveillance System (BRFSS) ACE module and other epidemiological surveys use ≥ 3 ACEs as a meaningful risk threshold (Center for Disease Control and Prevention (CDC), 2025).

### 2.4 Assessments

All behavioral assessments and questionnaires were administered in person or via videoconference by trained psychologists, who were native speakers of Brazilian Portuguese. Interviews were audiotaped for quality control. Using wireless tablets, interviewers entered data directly via an online computerized system (REDCap) designed to guarantee appropriate skips and correct coding, and prevent missing data. Supporting Information S1: Table 1 lists all assessments, when they were administered and who was interviewed/assessed. Detailed description of translation of assessments and data management is provided in the Supporting Information S1: Items 1.5 and 1.9.

### 2.5 Predictors

At the baseline assessment, maternal history of ACEs was retrospectively assessed with the CDC‐Kaiser ACE Study Questionnaire (Felitti et al. 2019), the Major Life Experiences (MLE) questionnaire (Turner and Lloyd 2004) and the Childhood Trauma Questionnaire (CTQ) (Bernstein et al. 2003; Grassi‐Oliveira et al. 2006). Detailed information on these instruments is available in the Supporting Information S1: Item 1.4.

Mothers were categorized into the high ACEs group if they endorsed three or more adverse experiences in the CDC‐Kaiser ACE Questionnaire administered on mothers' enrollment day. MLE was used for a more in‐depth approach to explore maternal exposure to a broader diversity of adverse events.

### 2.6 Outcomes

#### 2.6.1 Newborn Brain Imaging

**FIGURE 2:**
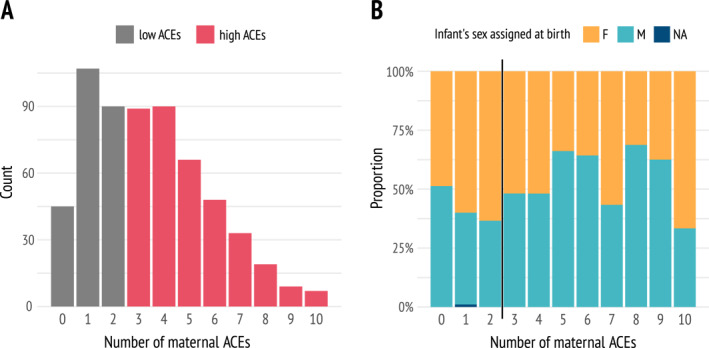
Profile of adverse childhood experiences (ACEs) and infant's biological sex in our sample. (A) Distribution of the number of ACEs reported by mothers (*n* = 603). Mothers reporting three or more ACEs in the CDC‐Kaiser ACE Questionnaire were categorized into the “high ACEs” group. (B) Proportion of infants' biological sex across the levels of maternal ACEs. The vertical line separates low (left) from high (right) maternal ACEs. F: female, M: male, NA: sex information not available.

After the clinical examination by a pediatrician at 2–4 weeks of age, newborns were scanned during natural sleep (no sedation) with a Siemens 3T MAGNETOM Skyra scanner using a 16‐channel head coil. Details of the newborn preparation for the exam are provided in the Supporting Information S1: Item 1.6 and Figure 2.

#### 2.6.2 Child Development and Behavior

Child development and behavior were evaluated with the Child Behavior Checklist (CBCL) 1½‐5 years (Achenbach and Rescorla 2000; Silvares et al. 2008), Ages and Stages third edition (ASQ‐3) (Filgueiras et al. 2013; Squires et al. 2009), Early Childhood Behavior Questionnaire—Very Short Form (ECBQ‐VSF) (Putnam et al. 2006), tasks from the Laboratory Temperament Assessment Battery (Lab‐TAB) (Goldsmith and Rothbart 1996a, 1996b) (Masks, Gentle Arm Restraint, Blocks, Dinky Toys, and Snack Delay), Behavior Inhibition tasks (stranger, truck, robot, and tunnel) adapted from (Tang et al. 2020), and the Bayley Scales of Infant Development third Edition (BSID‐3) (Bayley 2012; Madaschi et al. 2016).

Behavior during a brief mother‐child interaction was assessed once at baseline and then at all follow‐up visits. For this assessment, a mother‐child free‐play session lasting 5–7 min was videotaped and coded using the Coding Interactive Behavior (CIB) manual (Feldman 1998) and the Mind Mindedness Coding Manual version 2.2 (Meins and Fernyhough 2015). Coders were blind to maternal ACEs status and were trained until they reached at least 85% interrater reliability with the trainers. For newborns, the videos were micro‐coded following the CIB Newborn Manual with the Behavioral Observation Research Interactive Software (BORIS) (Friard and Gamba 2016) and also coded following the Mind Mindedness Coding Manual. For videos of children aged 6 months or older, CIB global coding was used as recommended by the CIB Global Coding Manual.

### 2.7 Mediators

Inflammatory markers were analyzed in maternal peripheral blood and cord blood. Cortisol was measured from maternal and child hair samples at baseline and in saliva samples at baseline and follow‐up visits. Maternal and child salivary oxytocin was measured at the first postpartum visit and each subsequent visit. More information on sample collection and storage for these measures are provided in the Supporting Information S1

Maternal mood and mental health were assessed with the Edinburgh Postnatal Depression Scale (EPDS) (Cox et al. 1987), the Patient Health Questionnaire (PHQ‐9) (Kroenke et al. 2001), General Anxiety Disorder (GAD‐7) (Spitzer et al. 2006), and the Perceived Stress Scale (PSS) (Luft et al. 2007). The Family History Screener for Epidemiologic Studies (FHE) was also used to screen mothers and their family members for psychiatric disorders (Lish et al. 1995).

### 2.8 Covariates and Additional Measurements

In the Supporting Information S1: Item 1.7, we provide a detailed description of how we assessed parent‐child conflicts, quality of home environment, maternal substance consumption, food security, maternal personality, experiences related to the COVID‐19 pandemic (COVEX), and socioeconomic status.

### 2.9 Biospecimens

**FIGURE 3:**
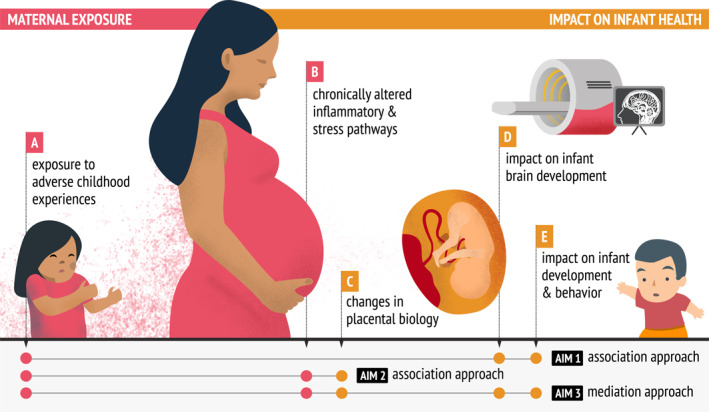
Visual description of the analysis plan of healthy MiNDS.

Biospecimens were collected during pregnancy, labor and postnatally from mothers and their children (Figure 1). Maternal biospecimens were collected during pregnancy (hair and blood), at labor (blood), at the MRI visit (saliva), and during follow‐up visits (saliva). Child biospecimens were obtained immediately after labor (cord blood and placenta), at the MRI visit (hair and saliva), and during follow‐up visits (saliva). A detailed description of the collection, processing, and storage procedures is provided in the Supporting Information S1: Item 1.8 and Figure 3.

## 3 Results

As of the date of the preparation of this manuscript, *Healthy MiNDs* is an ongoing study. Below we provide a short description of the cohort with numbers reflecting our sample in December 2024.

### 3.1 Enrollment and Follow‐Up Status

Between December 2020 and January 2024, 626 mothers were enrolled, of whom 603 completed the ACEs questionnaire. From these mothers, 547 mother‐child dyads were successfully enrolled. As of December 2024, 264 dyads (48%) remain active, and 133 (24%) have completed the final 24‐month follow‐up (Table 1).

**TABLE 1:** Number of enrolled participants, collected samples and follow‐ups performed.

	Count (%)
Pregnant women enrolled	626
Mother‐child dyads enrolled	547
Newborn MRI scans performed	303 (55%*^a^*)
Women with blood samples collected (at 3^rd^ trimester of gestation)	578 (92%*^b^*)
Women with hair sample collected (at 3^rd^ trimester of gestation)	602 (96%*^b^*)
Women with blood samples collected (at childbirth)	331 (61%*^b^*)
Children with cord blood samples collected	306 (61%*^a^*)
Children with hair sample collected (at 2–4 weeks of age)	353 (65%*^a^*)
Placenta samples collected	333 (61%*^a^*)
6‐month follow‐ups performed	263 (48%*^a^*)
14‐month follow‐ups performed (on‐going)^c^	232 (42%*^a^*)
18‐month follow‐ups performed (on‐going)^c^	174 (32%*^a^*)
24‐month follow‐ups performed (on‐going)^c^	133 (24%*^a^*)

^a^ in reference to the total number of dyads enrolled.

^b^ in reference to the total number of pregnant women enrolled.

^c^ as of December, 2024.

Of the 79 mothers who did not form dyads, the reasons were: loss to follow‐up (57%), voluntary withdrawal (33%), newborn ineligibility at birth (7%), and post‐enrollment maternal ineligibility (3%) (Supporting Information S1: Figure 1). Additionally, 192 dyads (35%) included at least one secondary caretaker during the 6 or 18‐month visits, with details of secondary caretaker‐infant relationships presented in Supporting Information S1: Table 3.

The baseline and 6‐month follow‐up phases are complete, and the remaining follow‐ups are expected to be concluded by January 2026. Currently, 23, 73, and 138 active dyads are eligible for the 14, 18, and 24‐month visits, respectively.

### 3.2 Maternal ACEs and Sample Demographics

Of the 603 mothers who completed the ACEs questionnaire, 361 (60%) reported high ACEs exposure (≥ 3 ACEs), based on the CDC‐Kaiser ACE Questionnaire (Figure 2A). Nearly 90% of enrolled families were classified into low socioeconomic strata (C or D‐E), with 34% falling into the lowest stratum (D‐E). Additionally, only 15.3% of mothers reported attending college, regardless of degree completion (Table 2). This highlights the significant social and economic disadvantage of the sample, reflecting limited access to educational and financial resources.

**TABLE 2:** Overall sample demographics.

	*N*	Overall
Mother's age	603	
Mean ± SD		26.44 ± 5.30
Range		18–38
CDC‐kaiser ACEs endorsed^∞^	603	
Median (interquartile range)		3 (0–1–5–10)
Range		0–10
CTQ total score	597	
Mean ± SD		39.56 ± 13.61
Range		25–101
Socioeconomic status^a^	532	
A1		4 (0.8%)
B1		4 (0.8%)
B2		48 (9.0%)
C1		102 (19.1%)
C2		193 (36.3%)
D‐E		181 (34.0%)
Mother's education	599	
Primary education		96 (7.9%)
Secondary education		452 (76.8%)
Post‐secondary education		51 (15.3%)
Mother's self‐reported race	602	
Asian		57 (9.5%)
Black		107 (17.8%)
Indigenous		1 (0.1%)
Multiracial		230 (38.2%)
White		207 (34.4%)
Enrollment site	600	
São Paulo		463 (77.2%)
Guarulhos		137 (22.8%)
Newborn's sex assigned at birth	539	
Female		276 (51.2%)
Male		263 (48.8%)
Newborn's race^b^	356	
Asian		4 (1.1%)
Black		24 (6.7%)
Indigenous		0 (0.0%)
Multiracial		163 (45.8%)
White		165 (46.4%)
Newborn's weight at birth (kg)	340	
Mean ± SD		3.28 ± 0.43
Range		2.07–4.50
Newborn's 5‐min Apgar	331	
Median (interquartile range)		9 (7–9–10–10)
Range		7–10
Delivery type	344	
Vaginal		209 (60.8%)
C‐section		131 (38.1%)
Forceps		4 (1.1%)
Gestational age at birth (wk)	536	
Mean ± SD		39.45 ± 1.26
Range		29.0–43.7

*Note:* Only mothers with ACEs report available were included in this table (*n* = 603). All infants enrolled were included (n = 547).

Abbreviations: CTQ: Childhood Trauma Questionnaire. *N*: number of non‐missing values. SD: standard deviation. wk: weeks.

^a^ Socioeconomic strata are ordered from the highest income (A1) to the lowest income (D‐E).

^b^ as reported reported by the mother.

When comparing mothers with low versus high ACEs exposure, no significant differences were observed in maternal age, socioeconomic status, education level, or self‐reported race (Supporting Information S1: Table 2). However, the group with high maternal ACEs exposure had newborns with lower median 5‐min Apgar scores (F_1,329_ = 13.25, *p* < 0.01) and a higher proportion of male newborns (χ^2^_1_ = 8.63, *p* < 0.01) (Figure 2B and Supporting Information S1: Table 2).

## 4 Discussion

Here we describe the methods and rationale for Healthy MiNDS, a cohort designed to explore how maternal ACEs relate to prenatal inflammation, stress, placental biology, and offspring brain‐behavior development in families from a low‐resource setting in a low‐middle‐income country (LMIC).

The study aims to examine the fetal programming hypothesis in a context where families face high levels of adversities. We hypothesize that maternal ACEs lead to biochemical changes in the mother—such as altered inflammatory and stress responses—that persist into adulthood. These changes affect the maternal environment during pregnancy (e.g., increased cytokines and cortisol), influencing fetal brain development and later child behaviors. Healthy MiNDS has been designed to investigate these mechanisms.

While the fetal programming hypothesis has garnered strong support, few studies have explored alternative pathways or mechanisms in detail. Existing research in humans linking fetal programming to offspring psychiatric risk has significant limitations, such as limited early infant neurodevelopment phenotyping (Lugo‐Candelas et al. 2018), a gap addressed by Healthy MiNDS. In line with few other studies available (Hendrix et al. 2021; Lugo‐Candelas et al. 2023; Moog et al. 2018), we assess neurodevelopment before school age with MRI scans acquired before newborns are 6 weeks old, facilitating the disentangling of prenatal effects from those of the postnatal environment.

We also explore the association between maternal ACEs, inflammation, and stress markers during pregnancy by collecting maternal serum at the second and third trimester. At childbirth, we also collect maternal and cord blood serum to measure inflammatory markers. These data allow us to test the association of maternal ACEs with adult inflammatory and stress markers, while integrating them with other measurements of our study.

We also investigate molecular mechanisms in the placenta, a key interface between mother and fetus that responds to maternal stress and inflammation, potentially transmitting maternal influences to the fetus (Bronson and Bale 2014; Kim et al. 2015). By studying placental gene expression and epigenetic modifications, we aim to identify biological processes involved in transmitting adversity across generations. These molecular signatures may also serve as biomarkers for identifying individuals at risk and help guide the development of targeted interventions to mitigate the effects of ACEs on the offspring.

We have structured our analysis plan into three aims (Figure 3). Aim 1 explores the associations between maternal ACEs and offspring brain‐behavior development using infant MRI scans and behavioral assessments of cognitive control. Aim 2 examines associations linking prenatal maternal inflammation, DNA methylation, and gene expression in the placenta. Aim 3 will focus on how maternal ACEs influence infant cognitive behaviors, investigating the mediating role of maternal inflammatory markers and placental epigenetics.

While the impact of ACEs has been widely studied in the US and parts of Europe, less is known about their impact in other settings. Greater São Paulo, with over 20 million inhabitants (Instituto Brasileiro de Geografia e Estatística (IBGE) 2023), experiences high levels of violence, particularly in the cities of São Paulo and Guarulhos. These cities have the highest numbers of homicides in the state, ranking among the 2.1% most violent cities in Brazil, with homicide rates of 13.2 and 19.6 per 100,000 inhabitants, respectively (Instituto de Pesquisa Econômica Aplicada (Ipea) 2019). The region also has high rates of impulsivity‐related conditions (e.g. ADHD, ODD, CD and substance use disorder) (Fleitlich‐Bilyk and Goodman 2004; Polanczyk et al. 2015; Quintana et al. 2015) and psychosocial adversities (Ribeiro et al. 2009, 2013). Despite these issues, no intergenerational research has examined the link between parental childhood adversities to these conditions in the area.

Conducting this study in Brazil enhances the generalizability of research on the intergenerational effects of ACEs across varying levels of deprivation and resources. It also provides clinical, genetic, and epigenetic data from a Brazilian sample, which has a highly admixed genetic background and is underrepresented in the existing literature (Breeze et al. 2022; Sirugo et al. 2019).

Our study was conducted within the SUS, one of the world's largest universal health care systems, providing free care to all in Brazil. SUS is widely used, especially by those in low‐income areas with high levels of violence (Porto et al. 2011). According to the National Health Survey (Pesquisa Nacional de Saúde – PNS 2019), the SUS provided prenatal care to about 70% of the pregnant women in 2013 and 2019, offering seven prenatal appointments and two ultrasound exams per pregnancy through Primary Health Care centers linked to maternity hospitals.

In summary, our study explores the biological mechanisms underlying the fetal programming hypothesis, combining extensive maternal clinical assessments with prenatal and postnatal inflammation and stress markers, placental DNA methylation, RNA/miRNA profiles, mother and child genotyping, postnatal levels of saliva oxytocin and cortisol, newborn MRI scans, and child behavioral data.

To control for confounders, we enroll participants from neighboring Primary Health Care centers, ensuring exposure to similar levels of community violence. We also account for maternal mental health, disability, social support, partner support, postnatal parental mood symptoms, and children's sex assigned at birth and nutrition.

Demographic analyses show that enrolled mothers are predominantly from low socioeconomic strata, making them highly vulnerable to adverse experiences. Consistent with another Brazilian cohort (Soares et al. 2016), we observed a high prevalence of ACEs, with 93% of mothers reporting at least one ACE. Of the 545 mother‐child dyads enrolled, we successfully acquired 303 MRI scans of infants at 2–6 weeks of age, achieving 95% of our target. We expect to assess 271 infants at the 24‐month follow‐up, corresponding to 85% of our goal.

The distribution of families across low (40%) and high (60%) ACEs groups is close to our anticipated 50‐50 split, ensuring good representation. We noticed a small but statistically significant difference of Apgar scores between groups, with median scores of 9 and 10 for the low and high ACEs groups, respectively. Both scores fall within the normal range (7–10), indicating no immediate clinical concerns. We also observed a higher frequency of male newborns in the high ACEs group. Although stress and ACEs typically do not affect offspring sex ratio, one study with a small sample of 225 women found that those exposed to two or more childhood traumas were more likely to give birth to female offspring (Kaitz et al. 2014). However, these findings should be interpreted with caution, as the generalizability may be limited by the study's Israeli sample, which could influence the prevalence of traumas or symptoms. Additionally, women with risky health behaviors, chronic health conditions, serious pregnancy complications, mood or anxiety disorders (excluding PTSD with or without MDD), as well as offspring who were either not born or born prematurely were excluded. This remains the only study reporting infant sex differences related to ACEs, highlighting the need for further research.

Our study has some limitations. Maternal ACEs were assessed through self‐report in adulthood. Although retrospective reports of ACEs are generally consistent with prospective ones (Reuben et al. 2016), factors such as avoidance of painful memories, current emotional state, and personality traits (e.g., agreeableness and neuroticism) can influence recall (Reuben et al. 2016). To address these concerns, we use current PTSD avoidance symptoms and indicators of anxiety and depression. We also assess neuroticism and agreeableness using the Ten‐Item Personality Inventory (TIPI) (Nunes et al. 2018). These measures help account for potential retrospective report biases in ACEs reporting. Additionally, as recommended (Reuben et al. 2016), Healthy MiNDS includes objectively measured offspring outcomes.

Fetal programming is not mutually exclusive with other mechanisms of intergenerational transmission, which are beyond the scope of our study. For instance, by influencing maternal behavior, maternal ACEs may affect not‐captured postnatal parent‐child interactions and thereby affect child neurodevelopment. Our study does not address mechanisms involving oocytes or sperm either.

In conclusion, Healthy MiNDS offers valuable insights into how maternal adverse childhood experiences (ACEs) influence child brain and behavioral development. By uncovering the underlying mechanisms, this study can inform targeted interventions to reduce the intergenerational transmission of adversity. For example, dietary or psychosocial strategies appropriate for low‐resource settings could be developed to reduce inflammation and protect neurodevelopment. If our mechanistic hypotheses are supported, future intervention studies could include clinical trials in which pregnant women with a history of ACEs are randomized to receive anti‐inflammatory interventions or treatment as usual. Ultimately, this line of research has the potential to inform scalable, evidence‐based strategies that promote healthier developmental trajectories for the next generation.

## Author Contributions

**Danilo Micali:** data curation, formal analysis, investigation, methodology, project administration, software, validation, visualization, writing – original draft, writing – review and editing. **Ana Carolina Coelho Milani:** investigation, methodology, writing – review and editing. **Camilla Salmeron:** investigation, methodology, resources. **Célia Araújo:** investigation, methodology, writing – review and editing. **Aline Camargo Ramos:** investigation, methodology, writing – review and editing. **Marcos Roberto Fanton:** investigation, methodology, writing – review and editing. **Sara B. VanBronkhorst:** funding acquisition, methodology, writing – review and editing. **Nitamar Abdala:** methodology, resources, writing – review and editing. **Ivaldo Silva:** conceptualization, funding acquisition, methodology, resources, supervision, writing – review and editing. **Sintia Iole Belangero:** conceptualization, funding acquisition, methodology, resources, supervision, writing – review and editing. **Cristiane S. Duarte:** conceptualization, funding acquisition, methodology, project administration, resources, supervision, writing – review and editing. **Jonathan Posner:** conceptualization, funding acquisition, methodology, project administration, resources, supervision, writing – review and editing. **Andrea Parolin Jackowski:** conceptualization, funding acquisition, methodology, project administration, resources, supervision, writing – review and editing.

## Ethics Statements

In Brazil, the study was approved by Comissão Nacional de Ética em Pesquisa (CONEP, National Commission of Ethics in Research, 78018417.2.0000.5505). In the United States, institutional review boards (IRBs) of both participating sites have approved this study (NYSPI IRB: 7927; Duke Health IRB: Pro00110664).

In accordance with CONEP guidelines, no compensation or financial incentives were provided to participants throughout the study, except for reimbursement of transportation expenses when attending neuroimaging sections or other behavioral assessments in our laboratory facilities.

## Consent

In accordance with the principles outlined in the Helsinki Declaration, written informed consent was obtained from eligible women after they had read and understood the participant information sheet. Newborns were considered enrolled only after confirmation of mothers' interest in continuing to participate in the study during hospital admission for childbirth. Ethics approval and consent to participate.

## Conflicts of Interest

The authors declare that they have no competing interests.

## Supporting information

Supporting Information S1

## Data Availability

The datasets generated during the current study are available in an anonymized version through the NIMH Data Archive repository (nda.nih.gov), under collection C3811.
