# Supplementary material for: The Intergenerational Impact of Maternal Childhood Adversity on Child Behavior and Neurodevelopment: The Healthy MiNDS Protocol
Source: Int J Methods Psychiatr Res. 2025 Jul 19;34(3):e70031. doi: 10.1002/mpr.70031 (PMC12275983; doi:10.1002/mpr.70031)
Supplement: Supplementary file 1 — Supporting Information S1 [file MPR-34-e70031-s001.pdf]

The intergenerational impact of maternal childhood adversity on child behavior and neurodevelopment: the Healthy MINDS protocol

Danilo Micali[1,2] | Ana Carolina Coelho Milani[3,4] | Camilla Salmeron[7] | Célia Araújo[1,3] | Aline Camargo Ramos[1,3] | Marcos Fanton[1,3] | Sara B. VanBronkhorst[5] | Nitamar Abdala[6] | Ivaldo Silva[7,9] | Síntia Iole Belangero[1,2,3] | Cristiane S. Duarte[5¶] | Jonathan Posner[8¶] | Andrea Parolin Jackowski[1,3,9¶*]

[1] Laboratory of Integrative Neuroscience (LiNC), Universidade Federal de São Paulo, São Paulo, Brazil | [2] Department of Genetics and Morphology, Universidade Federal de São Paulo, São Paulo, Brazil | [3] Department of Psychiatry, Universidade Federal de São Paulo, São Paulo, Brazil | [4] Department of Pediatrics, Universidade Federal de São Paulo, São Paulo, Brazil | [5] New York State Psychiatric Institute, Columbia University Irving Medical Center, New York, USA | [6] Department of Radiology, Universidade Federal de São Paulo, Brazil | [7] Department of Gynaecology, Universidade Federal de São Paulo, Brazil | [8] Duke University Medical Center, North Carolina, USA | [9] Department of Education, Information and Communications Technology (ICT) and Learning, Østfold University College, Halden, Norway.

TABLE OF CONTENTS

1 | SUPPLEMENTARY INFORMATION FOR 'METHODS' 2

1.1 | Enrollment 2
1.2 | Enrollment targets & power calculation 2
1.3 | Assessments 2
1.4 | ACE Study Questionnaire 4
1.5 | Translation of assessments 5
1.6 | Outcomes | Newborn brain imaging 5
1.7 | Covariates and additional measurements 5
1.8 | Biospecimens 6
1.9 | Data management 7
1.10 | Safety measures 7

2 | SUPPLEMENTARY INFORMATION FOR 'RESULTS' 8

2.1 | Sample demographics by maternal ACEs groups 8

3 | REFERENCES 10

4 | APPENDIX 11

1 | Supplementary Information for 'Methods'

1.1 | Enrollment

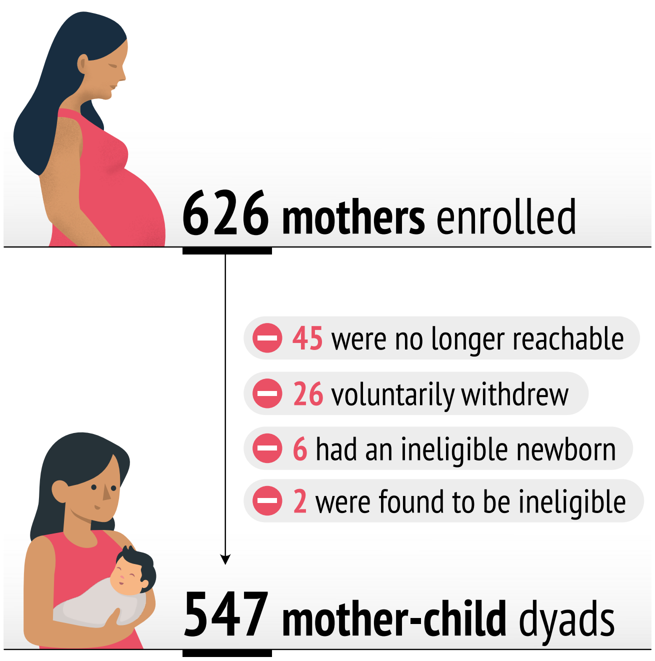

FIGURE S1 | Visual representation of the number of the enrolled mothers and reasons why 79 of them did not have their child also enrolled.

1.2 | Enrollment targets & power calculation

We estimated our sample size based on data from a pilot study with the same design and in a low-resource setting in São Paulo, Brazil (N = 62) conducted in 2018.

Aiming to have more than 80% power in all our analyses, we initially intended to screen 720 pregnant women in order to enroll 580 of them. Given the distribution of ACEs in São Paulo and Guarulhos, we expected the screening to yield around 50% in both high and low ACEs groups. With a cohort comprising 280 children, equally divided between low and high maternal ACEs exposure groups, based on two-sided tests at a significance level of 0.05, we would have over 80% power to detect differences in MRI measures between groups, with effect sizes of at least 0.34 considered medium. Utilizing ACEs as a continuous predictor, assuming a monotonic relationship, would enhance our statistical power further. Drawing from insights gained in our pilot study, where we observed a medium effect size association between functional connectivity and ACEs, we are optimistic about our ability to identify these associations.

After birth, we expected to obtain 400 infant MRI scans (around 70% of the 580 enrolled) and estimated that this would yield usable MRI data (e.g. no excessive head motion) in around 320 infants. We would continue to follow all 400 infants after the MRI session and estimated that we would retain 320 infants for the 24-month follow-up (80% of the 400 infants being followed).

To analyze inflammatory markers and placental gene expression/methylation, expecting detectable effect sizes stratified by infant sex with 80% power, at a medium effect size of 0.40, we expected to include 400 dyads. The power to detect mediation effects hinges on the size of correlations between maternal ACEs, pregnancy inflammatory markers, placental gene expression/DNA methylation, and infant MRI and cognitive control measures at 14- and 24-months. Through simulations with a sample size of 280, we find that if the effect size of ACEs on inflammatory markers and/or placental gene expression/methylation exceeds 0.26 SD (medium effect size), and if the association between inflammatory markers and/or placental gene expression/DNA methylation and MRI and cognitive control measures surpasses 0.26 SD (small to medium effect size), we possess over 80% power to detect mediation, whether through one or both mediators.

Finally, for the analysis of the impact of maternal ACEs on child behavior measures through longitudinal mixed effect regressions, we performed a power analysis based on conservative estimates, requiring a sample size of 320 children at the 24-month follow-up. Factoring in a moderate within-subject correlation ($r = 0.5$), we anticipate detecting cognitive control outcome differences of at least 0.32 standard deviation (SD) between high and low ACEs groups over time with more than 80% power at $\alpha = 0.05$.

1.3 | Assessments

The **Table S1** below lists all assessments used in the study, when they were administered and who was interviewed/assessed.

TABLE S1 | Description of measurements and timepoints of data collection

Topic	Instrument	Timepoint of assessment	Individual assessed	Respondent	Reference
Adverse Experiences	CDC-Kaiser ACEs Questionnaire	Baseline (prenatal)	M	M	(Felitti et al., 2019)
	Major Life Experiences	Baseline (prenatal)	M	M	(Turner & Lloyd, 2004)
	Childhood Trauma Questionnaire (CTQ)	Baseline (prenatal)	M	M	(Bernstein et al., 2003)
	Conflict Tactics Scale: Parent-Child Version	14M; 18M	M / C	M	(Reichenheim & Moraes, 2006)
Behavior & development	Mother-infant Interaction	Baseline (postpartum); 6M; 14M; 18M; 24M	M / C	M-C	(Feldman, 1998)
	Maternal-Fetal Attachment Scale (MFAS)	Baseline (prenatal)	M	M	(Garcia, 2021)
	Child Behavior Checklist (CBCL)	18M; 24M	C	M	(Achenbach & Rescorla, 2000; Silvares, Rocha, & Equipe Projeto Enurese, 2008)
	Ages & Stages Questionnaire 3rd Edition (ASQ-3)	14M; 24M	C	M	(Filgueiras, Pires, Maissonette, & Landeira-Fernandez, 2013; Squires, Bricker, Twonbly, & Potter, 2009)
	Early Childhood Behavior Questionnaire (ECBQ)	24M	C	M	(Putnam, Gartstein, & Rothbart, 2006)
	Laboratory Temperament Assessment Battery (Lab-TAB) – Masks	14M; 24M	C	C-behav	(Goldsmith & Rothbart, 1996a)
	Laboratory Temperament Assessment Battery (Lab-TAB) – Gentle Arm Restraint	6M; 18M	C	C-behav	(Goldsmith & Rothbart, 1996b, 1996a)
	Laboratory Temperament Assessment Battery (Lab-TAB) – Blocks, Dinky Toys, and Snack Delay	24M	C	C-behav	(Goldsmith & Rothbart, 1996a, 1996c)
	Behavior Inhibition Tasks	14M; 24M	C	C-behav	(Tang et al., 2020)
	Bayley Scales of Infant Development 3rd Edition (BSID-III)	14M; 24M	C	C-behav	(Bayley, 2012; Madaschi, Mecca, Macedo, & Paula, 2016)
Mood, Mental Health & Substance use	Edinburgh postnatal depression scale (EPDS)	Baseline (postpartum); 6M	M	M	(Cox, Holden, & Sagovsky, 1987)
	Patient Health Questionnaire (PHQ-9)	Baseline; 6M*; 14M; 18M*; 24M	M / S	M / S	(Kroenke, Spitzer, & Williams, 2001)
	General Anxiety Disorder (GAD-7)	Baseline; 6M*; 14M; 18M*; 24M	M / S	M / S	(Spitzer, Kroenke, Williams, & Löwe, 2006)

Topic	Instrument	Timepoint of assessment	Individual assessed	Respondent	Reference
	Perceived Stress Scale (PSS)	Baseline (prenatal); 6M*; 14M; 18M*; 24M	M / S	M / S	(Luft, Sanches, Mazo, & Andrade, 2007)
	Family History Screener for Epidemiologic Studies (FHE)	Baseline (postpartum)	M	M	(Lish, Weissman, Adams, Hoven, & Bird, 1995)
	Substance Use Questionnaire	Baseline (postpartum); 6M; 14M; 18M; 24M	M	M	–
	COVID-19 Experiences (COVEX)	Baseline	M	M	(Fisher et al., 2020)
Other measures	Brazilian Economic Classification Criteria 2019	Baseline (prenatal)	M	M	(ABEP, 2019)
	Brazilian Household Food Insecurity Scale	6M; 18M	M / C	M	(Segall-Corrêa & Marin-Leon, 2015)
	Home Observation for Measurement of the Environment	6M; 18M	M / C	C	(Cadwell & Bradley, 2016; Wendt, 2006)
	The Resilience Scale	Baseline (postpartum)	M	M	(Wagnild & Young, 1993)
	WHO Disability Assessment Schedule 2.0 (WHO-DAS)	Baseline (postpartum)	M	M	(Gold, 2014)

6M, 14M, 18M, 24M: 6-month, 14-month, 18-month and 24-month follow-ups, respectively. M: mother. C: child. S: significant other. C-behav: child's behavior. (*) indicates the timepoints in which significant others were assessed. "Baseline (prenatal)" refers to the period between mother enrollment (at 25-39th week of gestation) and childbirth. "Baseline (postpartum)" refers to the period of 2-6 weeks after childbirth.

1.4 | ACE Study Questionnaire

At the baseline assessment, maternal history of ACEs was retrospectively assessed with the CDC-Kaiser ACE Study Questionnaire (Felitti et al., 2019), the Major Life Experiences (MLE) (Turner & Lloyd, 2004) and the Childhood Trauma Questionnaire (CTQ) (Bernstein et al., 2003; Grassi-Oliveira, Stein, & Pezzi, 2006).

For the assessment of Adverse Childhood Experiences (ACEs) we used the ACE Study Questionnaire. Participants were queried about instances of verbal abuse, insults, degradation, or actions inducing fear of physical harm, as well as physical aggression like pushing, grabbing, slapping, or throwing objects, with consideration for resulting marks or injuries. The questionnaire also addressed experiences of sexual abuse, emotional neglect, and familial support, probing feelings of being unloved or unimportant within the family and the absence of mutual care, closeness, or support. Additionally, participants were asked about deprivation, including insufficient food, wearing dirty clothes, and lacking protection, as well as instances of parental separation or divorce. Domestic violence was investigated concerning physical aggression or threats with weapons toward a participant's mother or stepmother. Substance abuse within the household, mental health factors, and the impact of a household member going to prison were also assessed.

In the ACE Questionnaire, whenever endorsing an item, mothers were asked how often they had been exposed to that adversity and how old they were when it happened for the first and last time. For mothers who could not remember at what age or the school grade they were, we added the question "Was it before you were a teenager?" to help determine if the adversity happened during childhood or later.

For the MLE, we added three items extracted from the Survey of Exposure to Community Violence about "being verbally threatened to death", "being arrested and taken into custody", and "having one's house broken into". The answers to these three items followed the pattern originally used in MLE.

1.5 | Translation of assessments

Assessments not available in Brazilian Portuguese were translated according to the following process: forward translation, back-translation, review, and expert panel evaluation. The forward translator spoke both English and native Portuguese. Back-translation was performed by a translator who spoke both languages but had English as their native language. The review, conducted by another native speaker of English, was performed by comparing the semantics between the original version and the back-translation. The expert panel—able to speak both English and native Portuguese—analyzed the reviewer's notes and decided on the best approach for any discrepancies, aiming to guarantee cross-cultural and conceptual equivalence. This translation process was conducted for the CDC-Kaiser ACEs Questionnaire, MLE, FHE, the Ten-Item Personality Screener (TIPI), and the COVID-19 Experiences (COVEX).

1.6 | Outcomes | Newborn brain imaging

Before the MRI session, all newborns underwent a clinical examination by a pediatrician. Newborn's preparation for the exam is detailed in the Additional File. Newborns were then prepared for the MRI exam by wearing diapers, a t-shirt, ear protectors (ear plugs, wax, MRI Mini Muffs and a cap) and being swaddled in a blanket. An MRI safety screening questionnaire was filled out by the mother before she was taken to the MRI room with her newborn. The newborn was then breastfed and/or bottle-fed by the mothers while an oximeter was placed on the infant's feet to allow for monitoring of heart rate and oxygen saturation throughout the MRI scans. After falling into a natural sleep (no sedation was used), the newborn was positioned in the MRI machine with foams placed around their heads before setting the head coil. A visual summary of these procedures is shown in **Figure S2**.

MRI exams were performed at the Radiology Department of UNIFESP with a Siemens 3T MAGNETOM Skyra scanner using a 16-channel head coil. The MRI acquisition protocol was performed with the following parameters: (1) T2-weighted image parameters (TR = 3200, TE = 432, thickness = 0.99 mm, FOV = 190 mm, bandwidth = 723); (2) T1-weighted image parameters (TR = 2000 ms, TE = 2.8 ms, thickness = 0.99 mm, flip angle = 8°, FOV = 190 mm, bandwidth = 200, yielding 160 axial slices); (3) two resting functional MRI runs (TR = 2000, TE = 30.0, thickness = 3.0 mm, FOV = 192, bandwidth = 2520); and (4) a diffusion sequence (TR = 10700, TE = 91.0, thickness = 1,7 mm, FOV = 207, bandwidth = 1518).

A medical doctor and MRI technician were present for all MRI procedures. If the newborn woke during the scanning session, the mother could be called into the room for another round of breastfeeding and the exam continued if the newborn was able to fall asleep again. A report of the exam was provided by a radiologist to the newborn's family a few days later. Exams with incidental findings were analyzed by a child neurologist, who also provided mothers with proper counseling. As needed, the child was referred to Primary Health Care centers for medical follow-up.

1.7 | Covariates and additional measurements

In the follow-up visits, we used the CTS-PC (Conflict Tactics Scale: Parent-Child Version) to assess psychological/physical maltreatment and neglect of children by parents (Reichenheim & Moraes, 2006).

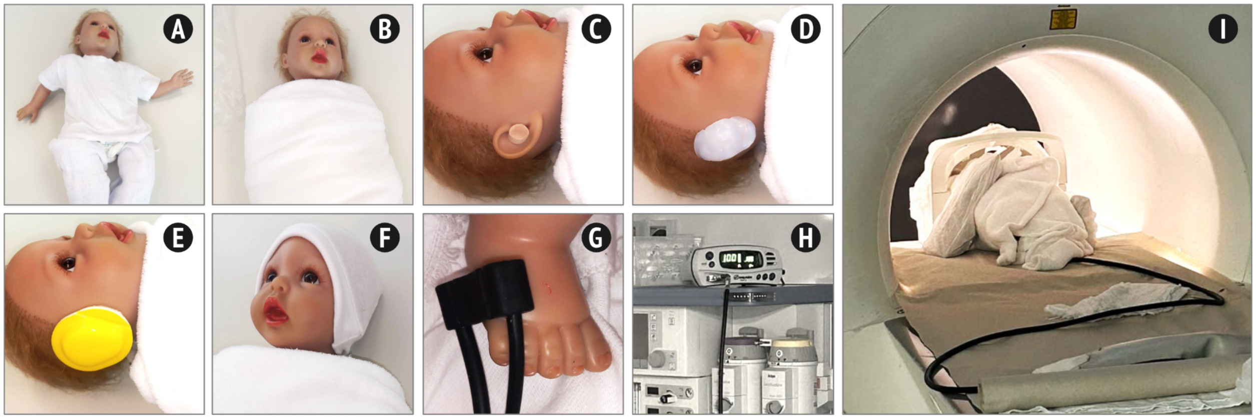

FIGURE S2 | Visual summary of the procedures for the newborn brain imaging scanning. (A) Putting on diapers and a t-shirt; (B) Wrapping in two swaddling clothes and blankets; (C) Placing ear plugs, (D) Placing ear wax; (E) Placing MRI Mini Muffs noise attenuators; (F) Putting on a head cap; (G) Placing the oximeter; (H) Monitoring of oxygen saturation and heart rate; (I) Closely monitored MRI scan.

The quality of the home environment was assessed by the Home Observation for Measurement of the Environment (HOME) - Infant/Toddler, with a reliability of at least 85% among the interviewers. As no official and validated translation of this assessment to Brazilian Portuguese was available to date, we adapted a previously translated version (Wendt, 2006) to match the items of the English version of HOME we selected for our study (Cadwell & Bradley, 2016).

The consumption of substances was assessed with a questionnaire developed by our team to collect data on the amount of tobacco, alcohol and other substances consumed by mothers before/during pregnancy [**Appendix**] and after childbirth. Questions were phrased as though the consumption of substances was expected for that timeframe, aiming to avoid mothers' embarrassment to report the use of substances during pregnancy. Instead of asking if the mother had consumed a given substance, items directly asked how much of that substance had been consumed. This questionnaire also included a section for a list of medicines used by mothers. For each medicine mentioned, mothers were asked if it had been used without a prescription or at dose different from prescribed. When this questionnaire was administered up to 6 weeks after childbirth, items addressed substance use for each trimester of pregnancy separately. At follow-up visits, items referred only to the period between visits.

Because the COVID-19 pandemic emerged during the course of the Healthy MINDS study, we used a shortened version of COVID-19 Experiences (COVEX) (Fisher et al., 2020) to assess mothers' experiences related to the COVID-19 outbreak. Items investigated COVID-19 symptoms or diagnoses, vulnerability to COVID-19, impact of the pandemic on living situation, worries or mental health changes, and concerns or difficulties related to the pregnancy.

Measures related to the quality of life were assessed with the Brazilian Food Insecurity Scale (Escala Brasileira de Medida da Insegurança Alimentar – EBIA) (Segall-Corrêa & Marin-Leon, 2015) and the WHO Disability Assessment Schedule 2.0 (WHO-DAS) (Gold, 2014). Maternal resilience was assessed during baseline visits by using the The Resilience Scale (Wagnild & Young, 1993).

Maternal personality is assessed using the Ten-Item Personality Screener (TIPI), a measure to be used to strengthen the retrospective measurement of ACEs.

Social economic status was determined by the Brazilian Economic Classification Criteria 2019 (ABEP, 2019) ranging from A (highest income) to D-E (lowest income). Both the instrument's estimate of the average household income for the assigned social economic stratum and the actual income informed by the mother were taken into account.

1.8 | Biospecimens

Biospecimens were collected during pregnancy, labor and postnatally from mothers and their children.

Maternal biospecimens. In the second and/or third trimester of gestation, peripheral blood was drawn from pregnant participants for serum isolation using Vacutainer® SST-II Advance® tubes (BD, USA). A hair sample was obtained for cortisol levels by cutting a thin interspaced strand of hair 0.5 cm to the scalp from the back of mothers' heads.

At labor, peripheral blood was drawn into two Vacutainer® EDTA tubes (BD), a PAXgene® Blood RNA tube (BD) and a Vacutainer® SST-II Advance® tube (BD) for the isolation of DNA, RNA, and serum, respectively. To guarantee sample quality, EDTA and PAXgene® Blood RNA tubes were collected in duplicate.

Maternal saliva was collected by the passive drool method at the baseline (postpartum), 6-month, 14-month and 24-month follow-up visits for oxytocin analysis and collected with OGR-575 saliva collection kits (DNA Genotek, Canada) at the 18-month visit for DNA analysis.

Child biospecimens. During labor, cord blood was collected and transferred into two Vacutainer® EDTA tubes, two PAXgene® Blood RNA tubes and a Vacutainer® SST-II Advance® tubes for isolation of DNA, RNA and serum, respectively. This collection was performed by using two regular 20-ml syringes with luer lock tips. The plunger was removed, and the umbilical cord was placed unclamped inside the barrel. After filling the barrel with cord blood, the plunger was repositioned, and the blood was immediately transferred through 18 G (1 1/2") needles from the syringes into blood collection tubes.

Placenta collection was performed up to 10 minutes after placental delivery to avoid sample degradation. Collection protocol was established according to recommendations previously described (Burton et al., 2014). The placenta was inspected for any abnormalities and membranes were removed to fully expose its fetal surface. From that surface, a sample of 5-10 mm3 of placental tissue was collected from 4 quadrants of the placenta, washed with sterile PBS 1x pH 7.4, and individually placed in 2-ml microtubes, each containing 1 ml of AllProtect Tissue Reagent (Qiagen, Germany) for immediate DNA, RNA and protein stabilization. Samples were kept for 12 hours at 4-8°C before being stored at -20°C. A collection kit was developed to enable and optimize placenta collection as shown on **Figure S3**.

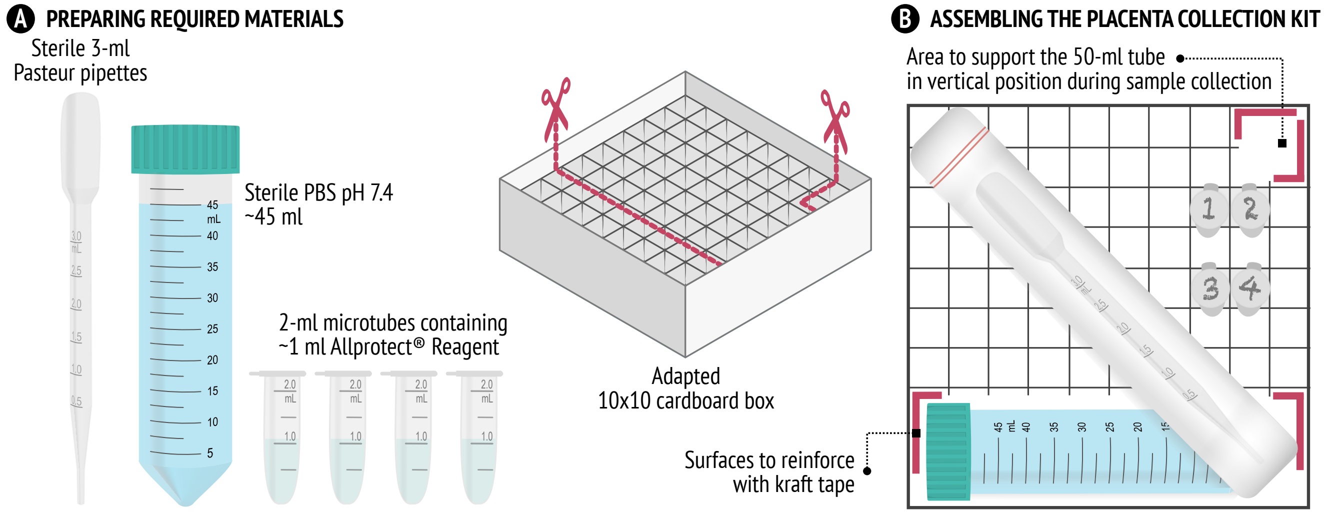

FIGURE S3 | Content and assembly of the placenta collection kit developed for Healthy MiNDS.

Child saliva was collected utilizing Salivettes® tubes at the baseline (postpartum), 6-month, 14-month and 24-month

follow-up visits and stored in the same manner as described above for maternal saliva collection. At 18-month visits, child saliva was also collected with Oragene OGR-575 saliva collection kits according to manufacturer's instructions.

Sample Processing. DNA was extracted from EDTA tubes with the Gentra® Puregene® Blood Kit (Qiagen, Germany). Total RNA (containing miRNAs) was isolated from PAXgene® Blood RNA tubes with the PAXgene® Blood miRNA kit (Qiagen).

For the placenta samples, approximately 7 mg of placental tissue was taken from each quadrant and pooled together (~30 mg), aiming to guarantee better representation of the whole organ. Then the pool was lysed with the TissueLyser LT (Qiagen) using 5-mm Stainless Steel Beads (Qiagen). DNA and RNA (containing miRNA) were isolated from the lysed pool with the AllPrep DNA/RNA/miRNA Universal kit. All protocols were executed according to the manufacturer's instructions.

Saliva samples (except OGR-575 kits) were stored at -20°C for one week, then were centrifuged for 15 minutes at 4000 rpm and stored at -80°C until they were analyzed.

Sample Storage. Isolated DNA and RNA were respectively stored in -20°C and -80°C freezers. Remaining placenta samples were kept submerged in AllProtect Tissue Reagent and stored under -20°C. Isolated serum samples were stored in -80°C in 200-µl aliquots. Hair samples (wrapped in foil sheets inside paper envelopes) were stored at room temperature. Aliquoted saliva samples were stored at -80°C and Oragene OGR-575 saliva collection kits were stored at room temperature. All samples were stored in the biorepository of the Laboratory of Integrative Neuroscience at UNIFESP.

1.9 | Data management

The study data was collected and managed by REDCap electronic data capture tools (Harris et al., 2019, 2009) hosted by Universidade Federal de São Paulo – Escola Paulista de Medicina, in Brazil. Access to personal health information was restricted to staff members in charge of participant enrollment and data collection. All MRI scan files generated were anonymized with the software DicomCleaner™ before being shared across sites or uploaded to public databases. All data and biospecimens were identified by a coded identification number.

1.10 | Safety measures

During the COVID-19 pandemic, all in-person protocols were carried out in accordance with COVID-19-related guidelines implemented by UNIFESP, PHC centers and maternity hospitals. During all procedures, staff members were reduced to a minimum and were equipped with disposable gloves, PFF2 masks and laboratory gowns. Participants were provided with hand sanitizer, new masks (changed every 2 hours or if wet) and a laboratory gown. Any material participants would be in touch with were either disposable (and immediately discarded after use) or properly disinfected (before and after use). When leaving, participants and staff members disposed of all safety equipment and were provided with new masks. Whenever local rates of COVID-19 infection were high, in-person protocols were performed only when strictly necessary or even fully suspended to guarantee the safety of participants and staff.

2 | Supplementary Information for 'Results'

2.1 | Sample demographics by maternal ACEs groups

TABLE S2 | Sample demographics by low vs high maternal ACEs groups

Variable	N	Low maternal ACEs (n = 242)	High maternal ACEs (n = 361)	Statistics
**Mother's age**	603			$F_{1,601} = 0.06$, p = 0.81 [2]
Mean ± SD		26.46 ± 5.22	26.43 ± 5.36	
Range		18–38	18–38	
CDC-Kaiser ACEs endorsed[∞]	603			$F_{1,601} = 1657.68$, **p < 0.01** [2]
Median (interquartile range)		1 (0–1–2–2)	5 (3–4–6–10)	
Range		0–2	3–10	
CTQ total score	597			$F_{1,595} = 300.12$, **p < 0.01** [2]
Mean ± SD		31.14 ± 6.09	45.22 ± 14.32	
Range		25–62	25–101	
Socioeconomic status[§]	532			$\chi^2_5 = 2.74$, p = 0.74 [1]
A1		3 (1.4%)	1 (0.3%)	
B1		1 (0.5%)	3 (1.0%)	
B2		19 (8.7%)	29 9.2%)	
C1		43 (19.7%)	59 (18.8%)	
C2		81 (37.1%)	112 (35.7%)	
D-E		71 (32.6%)	110 (35.0%)	
Mother's education	599			$\chi^2_2 = 0.39$, p = 0.82 [1]
Primary education		19 (7.9%)	32 (8,9%)	
Secondary education		185 (76.8%)	267 (74.6%)	
Post-secondary education		37 (15.3%)	59 (16.5%)	
Mother's self-reported race	602			$\chi^2_4 = 2.90$, p = 0.58 [1]
Asian		19 (7.9%)	38 (10.5%)	
Black		48 (19.8%)	59 (16.4%)	
Indigenous		0 (0.0%)	1 (0.3%)	
Multiracial		94 (38.8%)	136 (37.8%)	
White		81 (33.5%)	126 (35.0%)	
Enrollment site	600			$\chi^2_1 = 1.54$, p = 0.21 [1]
São Paulo		193 (79.8%)	270 (75.4%)	
Guarulhos		49 (20.2%)	88 (24.6%)	
Newborn's sex assigned at birth	539			$\chi^2_1 = 9.93$, **p < 0.01** [1]
Female		128 (59.5%)	148 (45.7%)	
Male		87 (40.5%)	176 (54.3%)	
Newborn's race*	356			$\chi^2_3 = 2.52$, p = 0.47 [1]
Asian		3 (2.2%)	1 (0.5%)	
Black		10 (7.3%)	14 (6.4%)	
Indigenous		0 (0.0%)	0 (0.0%)	
Multiracial		63 (46.0%)	100 (45.6%)	
White		61 (44.5%)	104 (49.5%)	
Newborn's weight at birth (kg)	340			$F_{1,338} = 0.29$, p = 0.59 [2]
Mean ± SD		3.26 ± 0.41	3.29 ± 0.43	
Range		2.07–4.47	2.33–4.50	

Variable	N	Low maternal ACEs (n = 242)	High maternal ACEs (n = 361)	Statistics
Newborn's 5-minute Apgar	331			$F_{1,329} = 13.25$, **$p < 0.01$** [2]
Median (interquartile range)		10 (7–9–10–10)	9 (7–9–10–10)	
Range		7–10	7–10	
Delivery type	344			$X^2_2 = 2.12$, $p = 0.35$ [1]
Vaginal		87 (65.4%)	122 (57.8%)	
C-section		45 (33.8%)	86 (40.8%)	
Forceps		1 (0.8%)	3 (1.4%)	
Gestational age at birth (wk)	536			$F_{1,534} = 0.05$, $p = 0.82$ [2]
Mean ± SD		39.50 ± 1.10	39.41 ± 1.36	
Range		34.7–43.7	29.0–43.4	

Only mothers with ACEs report available were included in this table (n=603). All infants enrolled were included (n=547). Significant p-values are presented in bold. **CTQ**: Childhood Trauma Questionnaire. **N**: number of non-missing values. **SD**: standard deviation. **wk**: weeks. [1]Pearson. [2]Wilcoxon. [∞]women who reported 3 or more ACEs. [§]Socioeconomic status categories are ordered from the highest income (A1) to the lowest income (D-E). *as reported reported by the mother

TABLE S3 | Relationship of second caretakers with enrolled infants

	Count (%)
**Second caretaker enrolled at the 6-month visit**	**154 (100%)**
Father, father-in-law, male partner of biological mother	112 (72.7%)
Mother, mother-in-law, female partner of biological mother	1 (0.6%)
Siblings	3 (1.9%)
Aunts/Uncles	7 (4.5%)
Cousins	1 (0.6%)
Grandparents	27 (17.5%)
Babysitter or another caretaker (not a relative)	2 (1.3%)
Other	5 (3.2%)
**Second caretaker enrolled at the 18-month visit**	**113 (100%)**
Father, father-in-law, male partner of biological mother	72 (63.7%)
Mother, mother-in-law, female partner of biological mother	0 (0%)
Siblings	3 (2.7%)
Aunts/Uncles	7 (6.2%)
Cousins	0 (0%)
Grandparents	23 (20.4%)
Babysitter or another caretaker (not a relative)	1 (0.9%)
Other	7 (6.2%)

Relationships are described in reference to the infant.

3 | References

ABEP. (2019). Critério Brasil: Critério de Classificação Econômica Brasil 2019. Retrieved from https://www.abep.org/criterio-brasil

Achenbach, T. M., & Rescorla, L. A. (2000). Manual for the Aseba Preschool Forms & Profiles. Burlington, VT: University of Vermont, Research Center for Children, Youths, & Families.

Bayley, N. (2012). Bayley Scales of Infant and Toddler Development, Third Edition [Data set]. American Psychological Association. doi: 10.1037/t14978-000

Bernstein, D. P., Stein, J. A., Newcomb, M. D., Walker, E., Pogge, D., Ahluvalia, T., … Zule, W. (2003). Development and validation of a brief screening version of the Childhood Trauma Questionnaire. Child Abuse & Neglect, 27(2), 169–190. doi: 10.1016/S0145-2134(02)00541-0

Burton, G. J., Sebire, N. J., Myatt, L., Tannetta, D., Wang, Y.-L., Sadovsky, Y., … Redman, C. W. (2014). Optimising sample collection for placental research. Placenta, 35(1), 9–22. doi: 10.1016/j.placenta.2013.11.005

Cadwell, B. M., & Bradley, R. H. (2016). Home Observation for Measurement of the Environment: Administration Manual. Tempe, AZ: Family & Human Dynamics Research Institute, Arizona State University.

Cox, J. L., Holden, J. M., & Sagovsky, R. (1987). Detection of Postnatal Depression: Development of the 10-item Edinburgh Postnatal Depression Scale. British Journal of Psychiatry, 150(6), 782–786. doi: 10.1192/bjp.150.6.782

Feldman, R. (1998). Coding interactive behavior manual.

Felitti, V. J., Anda, R. F., Nordenberg, D., Williamson, D. F., Spitz, A. M., Edwards, V., … Marks, J. S. (2019). REPRINT OF: relationship of childhood abuse and household dysfunction to many of the leading causes of death in adults: The adverse childhood experiences (ACE) study. American Journal of Preventive Medicine, 56(6), 774–786. doi: 10.1016/j.amepre.2019.04.001

Filgueiras, A., Pires, P., Maissonette, S., & Landeira-Fernandez, J. (2013). Psychometric properties of the Brazilian-adapted version of the Ages and Stages Questionnaire in public child daycare centers. Early Human Development, 89(8), 561–576. doi: 10.1016/j.earlhumdev.2013.02.005

Fisher, P. W., Desai, P., Klotz, J., Turner, J. B., Reyes-Portillo, J. A., Ghisolfi, I., … Duarte, C. S. (2020). COVID-19 Experiences (COVEX).

Garcia, M. C. (2021). Versão Brasileira da Escala de Apego Materno-Fetal (MFAS) (Thesis). Universidade do Sul de Santa Catarina, Palhoça, Santa Catarina.

Gold, L. H. (2014). DSM-5 and the assessment of functioning: The World Health Organization Disability Assessment Schedule 2.0 (WHODAS 2.0). The Journal of the American Academy of Psychiatry and the Law, 42(2), 173–181.

Goldsmith, H. H., & Rothbart, M. K. (1996a). The Laboratory Temperament Assessment Battery (LAB-TAB): Locomotor [Technical Report]. Madison, WI, USA: Psychology Department, University of Wisconsin.

Goldsmith, H. H., & Rothbart, M. K. (1996b). The Laboratory Temperament Assessment Battery (LAB-TAB): Prelocomotor [Technical Report]. Madison, WI, USA: Psychology Department, University of Wisconsin.

Goldsmith, H. H., & Rothbart, M. K. (1996c). The Laboratory Temperament Assessment Battery (LAB-TAB): Preschooler [Technical Report]. Madison, WI, USA: Psychology Department, University of Wisconsin.

Grassi-Oliveira, R., Stein, L. M., & Pezzi, J. C. (2006). Tradução e validação de conteúdo da versão em português do Childhood Trauma Questionnaire. Revista de Saúde Pública, 40(2), 249–255. doi: 10.1590/S0034-89102006000200010

Harris, P. A., Taylor, R., Minor, B. L., Elliott, V., Fernandez, M., O'Neal, L., … REDCap Consortium. (2019). The REDCap consortium: Building an international community of software platform partners. Journal of Biomedical Informatics, 95, 103208. doi: 10.1016/j.jbi.2019.103208

Harris, P. A., Taylor, R., Thielke, R., Payne, J., Gonzalez, N., & Conde, J. G. (2009). Research electronic data capture (REDCap)—A metadata-driven methodology and workflow process for providing translational research informatics support. Journal of Biomedical Informatics, 42(2), 377–381. doi: 10.1016/j.jbi.2008.08.010

Kroenke, K., Spitzer, R. L., & Williams, J. B. W. (2001). The PHQ-9: Validity of a brief depression severity measure. Journal of General Internal Medicine, 16(9), 606–613. doi: 10.1046/j.1525-1497.2001.016009606.x

Lish, J. D., Weissman, M. M., Adams, P. B., Hoven, C. W., & Bird, H. (1995). Family psychiatric screening instrument for epidemiologic studies: Pilot testing and validation. Psychiatry Research, 57(2), 169–180. doi: 10.1016/0165-1781(95)02632-7

Luft, C. D. B., Sanches, S. D. O., Mazo, G. Z., & Andrade, A. (2007). Versão brasileira da Escala de Estresse Percebido: Tradução e validação para idosos. Revista de Saúde Pública, 41(4), 606–615. doi: 10.1590/S0034-89102007000400015

Madaschi, V., Mecca, T. P., Macedo, E. C., & Paula, C. S. (2016). Bayley-III Scales of Infant and Toddler Development: Transcultural Adaptation and Psychometric Properties. Paidéia (Ribeirão Preto), 26(64), 189–197. doi: 10.1590/1982-43272664201606

Putnam, S. P., Gartstein, M. A., & Rothbart, M. K. (2006). Measurement of fine-grained aspects of toddler temperament: The early childhood behavior questionnaire. Infant Behavior & Development, 29(3), 386–401. doi: 10.1016/j.infbeh.2006.01.004

Reichenheim, M. E., & Moraes, C. L. (2006). Psychometric properties of the Portuguese version of the Conflict Tactics Scales: Parent-child Version (CTSPC) used to identify child abuse. Cadernos de Saúde Pública, 22(3), 503–515. doi: 10.1590/S0102-311X2006000300005

Segall-Corrêa, A. M., & Marin-Leon, L. (2015). A segurança alimentar no Brasil: Proposição e usos da escala brasileira de medida da insegurança alimentar (EBIA) de 2003 a 2009. Segurança Alimentar e Nutricional, 16(2), 1–19. doi: 10.20396/san.v16i2.8634782

Silvares, E. F. M., Rocha, M. M., & Equipe Projeto Enurese. (2008). Versão brasileira não publicada do inventário "Child Behavior Checklist for ages 1 1/2-5".

Spitzer, R. L., Kroenke, K., Williams, J. B. W., & Löwe, B. (2006). A Brief Measure for Assessing Generalized Anxiety Disorder: The GAD-7. Archives of Internal Medicine, 166(10), 1092. doi: 10.1001/archinte.166.10.1092

Squires, J., Bricker, D. D., Twonbly, E., & Potter, L. (2009). Ages & Stages Questionnaires: A parent-completed child monitoring system (3rd ed.). Baltimore: Paul H. Brookes Publishing.

Tang, A., Crawford, H., Morales, S., Degnan, K. A., Pine, D. S., & Fox, N. A. (2020). Infant behavioral inhibition predicts personality and social outcomes three decades later. Proceedings of the National

Academy of Sciences, 117(18), 9800–9807. doi: 10.1073/pnas.1917376117

Turner, R. J., & Lloyd, D. A. (2004). Stress Burden and the Lifetime Incidence of Psychiatric Disorder inYoung Adults: Racial and Ethnic Contrasts. Archives of General Psychiatry, 61(5), 481. doi: 10.1001/archpsyc.61.5.481

Wagnild, G. M., & Young, H. M. (1993). Development and psychometric evaluation of the Resilience Scale. Journal of Nursing Measurement, 1(2), 165–178.

Wendt, N. C. (2006). Fatores de risco e de proteção para o desenvolvimento da criança durante a transição para a parentalidade (Thesis, Universidade Federal de Santa Catarina). Universidade Federal de Santa Catarina, Florianópolis, SC. Repositório Institucional da UFSC. Retrieved from api.core.ac.uk/oai/oai:repositorio.ufsc.br:123456789/88679

4 | Appendix

In the next pages we provide the questionnaire developed by our team to assess the consumption of tobacco, alcohol and other substances consumed by mothers before/during pregnancy and after childbirth. The questionnaire used was in Brazilian Portuguese, however, we also provide a version of questionnaire in English.

QUESTIONNAIRE OF SUBSTANCE USE DURING PREGNANCY

Interviewer ______________________________ **Date of Assessment** ____________________

Participant ID ________________ **Participant Initials** ____________

SECTION 1: CIGARETTES AND ALCHOOL

	(a) 3 months **before** pregnancy	(b) 1st trimester of pregnancy	(c) 2nd trimester of pregnancy	(d) 3rd trimester of pregnancy	(e) Current (from baby birth until now)
**1** How many cigarettes (per day/week/month) do/did you usually smoke on average in each period?	________ cigarettes per ☐ day ☐ week ☐ month ☐ Refused ☐ Don't know	________ cigarettes per ☐ day ☐ week ☐ month ☐ Refused ☐ Don't know	________ cigarettes per ☐ day ☐ week ☐ month ☐ Refused ☐ Don't know	________ cigarettes per ☐ day ☐ week ☐ month ☐ Refused ☐ Don't know	________ cigarettes per ☐ day ☐ week ☐ month ☐ Refused ☐ Don't know
**2** How many people **currently** living with your baby smoke (including yourself)?					________ people
**3** How many doses of alcoholic drinks (per day/week/month) do/did you usually have on average in each period? *[see reference for doses bellow]*	________ doses per ☐ day ☐ week ☐ month ☐ Refused ☐ Don't know	________ doses per ☐ day ☐ week ☐ month ☐ Refused ☐ Don't know	________ doses per ☐ day ☐ week ☐ month ☐ Refused ☐ Don't know	________ doses per ☐ day ☐ week ☐ month ☐ Refused ☐ Don't know	________ doses per ☐ day ☐ week ☐ month ☐ Refused ☐ Don't know

SECTION 2: MEDICINES

[PROVIDE THE LIST OF SUBSTANCES #1 TO THE INTERVIEWEE]

During the past 12 months, which medicines did you use? Did you use any of them without a prescription or at a dose different than prescribed?

Instruction*: For each medicine used check the periods the participant used (“****Use****”) e if it was used without a prescription or at dose different from prescribed (“****NP/HD****”).*

	(a) 3 months <u>before</u> pregnancy		(b) 1st trimester of pregnancy		(c) 2nd trimester of pregnancy		(d) 3rd trimester of pregnancy		(e) Current (from birth to now)	
	Use	NP/HD	Use	HP/HD	Use	NP/HD	Use	NP/HD	Use	NP/HD
**4**	☐	☐	☐	☐	☐	☐	☐	☐	☐	☐
**5**	☐	☐	☐	☐	☐	☐	☐	☐	☐	☐
**6**	☐	☐	☐	☐	☐	☐	☐	☐	☐	☐
**7**	☐	☐	☐	☐	☐	☐	☐	☐	☐	☐
**8**	☐	☐	☐	☐	☐	☐	☐	☐	☐	☐
**9**	☐	☐	☐	☐	☐	☐	☐	☐	☐	☐
**10**	☐	☐	☐	☐	☐	☐	☐	☐	☐	☐
**11**	☐	☐	☐	☐	☐	☐	☐	☐	☐	☐
**12**	☐	☐	☐	☐	☐	☐	☐	☐	☐	☐
**13**	☐	☐	☐	☐	☐	☐	☐	☐	☐	☐
**14**	☐	☐	☐	☐	☐	☐	☐	☐	☐	☐

COMMENTS

SECTION 3: DRUGS (OTHER THAN ALCOHOL)

[PROVIDE THE LIST OF SUBSTANCES #1 TO THE INTERVIEWEE]

	(a) **3 months** <u>before</u> pregnancy	(b) **1st trimester** of pregnancy	(c) **2nd trimester** of pregnancy	(d) **3rd trimester** of pregnancy	(e) **Current** (from baby birth until now)
**15** Do/did you use drugs <u>other than alcohol</u> in any of these periods? →	☐ Yes<br>☐ No<br>☐ Refused<br>☐ Don't know	☐ Yes<br>☐ No<br>☐ Refused<br>☐ Don't know	☐ Yes<br>☐ No<br>☐ Refused<br>☐ Don't know	☐ Yes<br>☐ No<br>☐ Refused<br>☐ Don't know	☐ Yes<br>☐ No<br>☐ Refused<br>☐ Don't know
For every period checked YES in item 5, ask:					
**16** How many times on average (per day/week/month) do/did you use drugs other than alcohol in each period?	________<br>doses per<br>☐ day<br>☐ week<br>☐ month<br><br>☐ Refused<br>☐ Don't know	________<br>doses per<br>☐ day<br>☐ week<br>☐ month<br><br>☐ Refused<br>☐ Don't know	________<br>doses per<br>☐ day<br>☐ week<br>☐ month<br><br>☐ Refused<br>☐ Don't know	________<br>doses per<br>☐ day<br>☐ week<br>☐ month<br><br>☐ Refused<br>☐ Don't know	________<br>doses per<br>☐ day<br>☐ week<br>☐ month<br><br>☐ Refused<br>☐ Don't know
**17** Which type of drug other than alcohol do/did you use?<br><br>*[check all drugs provided by the interviewee]*	☐ Cannabis<br>☐ Amphetamines<br>☐ Ketamine<br>☐ Anabolic Substances<br>☐ Teas<br>☐ Cocaine<br>☐ Opioids<br>☐ Hallucinogens<br>☐ Solvents & Inhalants<br>☐ GHB & others	☐ Cannabis<br>☐ Amphetamines<br>☐ Ketamine<br>☐ Anabolic Substances<br>☐ Teas<br>☐ Cocaine<br>☐ Opioids<br>☐ Hallucinogens<br>☐ Solvents & Inhalants<br>☐ GHB & others	☐ Cannabis<br>☐ Amphetamines<br>☐ Ketamine<br>☐ Anabolic Substances<br>☐ Teas<br>☐ Cocaine<br>☐ Opioids<br>☐ Hallucinogens<br>☐ Solvents & Inhalants<br>☐ GHB & others	☐ Cannabis<br>☐ Amphetamines<br>☐ Ketamine<br>☐ Anabolic Substances<br>☐ Teas<br>☐ Cocaine<br>☐ Opioids<br>☐ Hallucinogens<br>☐ Solvents & Inhalants<br>☐ GHB & others	☐ Cannabis<br>☐ Amphetamines<br>☐ Ketamine<br>☐ Anabolic Substances<br>☐ Teas<br>☐ Cocaine<br>☐ Opioids<br>☐ Hallucinogens<br>☐ Solvents & Inhalants<br>☐ GHB & others

COMMENTS

LIST #1: MEDICINES

**BENZODIAZEPINES (tranquilizers)**	Diazepam, Rivotril®, Vallium®, Lexotan®, Olcadil®, Lorax®, Frontal®
**AMPHETAMINES (stimulants)**	Remédios para emagrecer, Ritalina®, Hipofagin®, Dualid®, Femproporex
**BARBITURATES (sedatives)**	Gardenal®, Hidantal®, fenobarbital
**ANABOLIC STEROIDS**	Winstrol®, Androxon®, Nebido®, Durateston®, Estandron®, Deca-durabolim®, Deposteron®, Testex®
**OPIATES (painkillers)**	Tylex®, Dolantina®, Codein®, Codex®
**ANTICHOLINERGICS**	Artane®, Akineton®, Atropina®
**KETAMINE**	Dopalen®

LIST #2: DRUGS (OTHER THAN ALCOHOL)

**CANNABIS**	Maconha, Haxixe, Óleo Skank
**AMPHETAMINES**	Remédio para ficar acordado (rebite)
**KETAMINE**	Special K, Super K
**TEAS**	Ayahuasca, Santo Daime
**COCAINE**	Crack Pó, Merla, Oxi, Pasta base, Folhas de Coca
**OPIOIDS**	Heroína, Heroína em fumo, Ópio
**HALLUCINOGENS**	Ecstasy, Bala, LSD, Mescalina, Peiote, PCP, Pó de anjo, Cristal, DMT
**SOLVENTS & INHALANTS**	Lança-perfume, Loló, Cola de sapateiro, Acetona, Thinner, Éter, Fluido de isqueiro
**GHB & OTHERS**	GHB, Boa noite cinderela, Droga do Estupro, Poppers (Nitrato de Amila), Gás do riso (Halotano)

Lists based on:
Fundação Oswaldo Cruz - Instituto de Comunicação e Informação Científica e Tecnológica em Saúde. *3rd National survey on drug use by the brazilian population*. 2017. Available at <https://www.arca.fiocruz.br/handle/icict/34614>.

QUESTIONÁRIO DE USO DE SUBSTÂNCIAS DURANTE A GESTAÇÃO

Entrevistador(a) ______________________________ **Data de aplicação** ____________________

ID da participante ____________________ **Iniciais da participante** ______________

SEÇÃO 1: CIGARRO E ÁLCOOL

	(a)	(b)	(c)	(d)	(e)
	**3 meses** antes da gestação	**1º trimestre** da gestação	**2º trimestre** da gestação	**3º trimestre** da gestação	**Atualmente** (do parto até hoje)
**1** Quantos cigarros (por dia/semana/mês) você fumou, em média, em cada período?	______ cigarros por ☐ dia ☐ semana ☐ mês ☐ Recusou ☐ Não sabe	______ cigarros por ☐ dia ☐ semana ☐ mês ☐ Recusou ☐ Não sabe	______ cigarros por ☐ dia ☐ semana ☐ mês ☐ Recusou ☐ Não sabe	______ cigarros por ☐ dia ☐ semana ☐ mês ☐ Recusou ☐ Não sabe	______ cigarros por ☐ dia ☐ semana ☐ mês ☐ Recusou ☐ Não sabe
**2** Quantas pessoas que atualmente moram com seu bebê fumam (incluindo você mesma)?					______ pessoa(s)
**3** Quantas doses de bebidas alcoólicas você costuma tomar (por dia/semana/mês) em média? *[veja referência para dose abaixo]*	______ doses por ☐ dia ☐ semana ☐ mês ☐ Recusou ☐ Não sabe	______ doses por ☐ dia ☐ semana ☐ mês ☐ Recusou ☐ Não sabe	______ doses por ☐ dia ☐ semana ☐ mês ☐ Recusou ☐ Não sabe	______ doses por ☐ dia ☐ semana ☐ mês ☐ Recusou ☐ Não sabe	______ doses por ☐ dia ☐ semana ☐ mês ☐ Recusou ☐ Não sabe

SEÇÃO 2: MEDICAMENTOS

[ENTREGUE A LISTA DE SUBSTÂNCIAS Nº 1 PARA A ENTREVISTADA]
Durante os últimos 12 meses, qual ou quais medicamentos você usou? Algum foi sem receita e/ou fora da dose recomendada?

> ***Instrução***: *Para cada medicamento utilizado marque os períodos em que se fez uso ("**Uso**") e se foi feito sem receita ou em dose diferente da receitada ("**SR/AD**").*

	(a) 3 meses antes de engravidar		(b) 1º trimestre de gestação		(c) 2º trimestre de gestação		(d) 3º trimestre de gestação		(e) Atual (desde que o bebê nasceu)	
	Uso	SR/AD	Uso	SR/AD	Uso	SR/AD	Uso	SR/AD	Uso	SR/AD
**4**	☐	☐	☐	☐	☐	☐	☐	☐	☐	☐
**5**	☐	☐	☐	☐	☐	☐	☐	☐	☐	☐
**6**	☐	☐	☐	☐	☐	☐	☐	☐	☐	☐
**7**	☐	☐	☐	☐	☐	☐	☐	☐	☐	☐
**8**	☐	☐	☐	☐	☐	☐	☐	☐	☐	☐
**9**	☐	☐	☐	☐	☐	☐	☐	☐	☐	☐
**10**	☐	☐	☐	☐	☐	☐	☐	☐	☐	☐
**11**	☐	☐	☐	☐	☐	☐	☐	☐	☐	☐
**12**	☐	☐	☐	☐	☐	☐	☐	☐	☐	☐
**13**	☐	☐	☐	☐	☐	☐	☐	☐	☐	☐
**14**	☐	☐	☐	☐	☐	☐	☐	☐	☐	☐

OBSERVAÇÕES

SEÇÃO 3: DROGAS (QUE NÃO ÁLCOOL)

[ENTREGUE A LISTA DE SUBSTÂNCIAS Nº 2 PARA A ENTREVISTADA]

	(a) 3 meses antes da gravidez	(b) 1º trimestre de gravidez	(c) 2º trimestre de gravidez	(d) 3º trimestre de gravidez	(e) Atual (desde que o bebê nasceu)
**15** Você usou drogas (sem ser álcool) nesses períodos? ➔	☐ Sim<br>☐ Não<br>☐ Recusou<br>☐ Não sabe	☐ Sim<br>☐ Não<br>☐ Recusou<br>☐ Não sabe	☐ Sim<br>☐ Não<br>☐ Recusou<br>☐ Não sabe	☐ Sim<br>☐ Não<br>☐ Recusou<br>☐ Não sabe	☐ Sim<br>☐ Não<br>☐ Recusou<br>☐ Não sabe
Para os períodos marcados com SIM no item anterior, pergunte:					
**16** Quantas vezes em média (por dia/semana/mês) você usa/usou essa(s) drogas em cada período?	________ vezes por<br>☐ dia<br>☐ semana<br>☐ mês<br>☐ Recusou<br>☐ Não sabe	________ vezes por<br>☐ dia<br>☐ semana<br>☐ mês<br>☐ Recusou<br>☐ Não sabe	________ vezes por<br>☐ dia<br>☐ semana<br>☐ mês<br>☐ Recusou<br>☐ Não sabe	________ vezes por<br>☐ dia<br>☐ semana<br>☐ mês<br>☐ Recusou<br>☐ Não sabe	________ vezes por<br>☐ dia<br>☐ semana<br>☐ mês<br>☐ Recusou<br>☐ Não sabe
**17** Qual tipo de droga você usa/usou?<br>*[marque todas que a entrevistada relatar]*	☐ *Cannabis*<br>☐ Anfetaminas<br>☐ Quetaminas<br>☐ Anabolizantes<br>☐ Chás<br>☐ Cocaína<br>☐ Opioides<br>☐ Alucinógenos<br>☐ Solventes ou inalantes<br>☐ GHB e outros	☐ *Cannabis*<br>☐ Anfetaminas<br>☐ Quetaminas<br>☐ Anabolizantes<br>☐ Chás<br>☐ Cocaína<br>☐ Opioides<br>☐ Alucinógenos<br>☐ Solventes ou inalantes<br>☐ GHB e outros	☐ *Cannabis*<br>☐ Anfetaminas<br>☐ Quetaminas<br>☐ Anabolizantes<br>☐ Chás<br>☐ Cocaína<br>☐ Opioides<br>☐ Alucinógenos<br>☐ Solventes ou inalantes<br>☐ GHB e outros	☐ *Cannabis*<br>☐ Anfetaminas<br>☐ Quetaminas<br>☐ Anabolizantes<br>☐ Chás<br>☐ Cocaína<br>☐ Opioides<br>☐ Alucinógenos<br>☐ Solventes ou inalantes<br>☐ GHB e outros	☐ *Cannabis*<br>☐ Anfetaminas<br>☐ Quetaminas<br>☐ Anabolizantes<br>☐ Chás<br>☐ Cocaína<br>☐ Opioides<br>☐ Alucinógenos<br>☐ Solventes ou inalantes<br>☐ GHB e outros

OBSERVAÇÕES

LISTA Nº1: MEDICAMENTOS

**TRANQUILIZANTES BENZODIAZEPÍNICOS**	Diazepam, Rivotril®, Vallium®, Lexotan®, Olcadil®, Lorax®, Frontal®
**ESTIMULANTES ANFETAMÍNICOS**	Remédios para emagrecer, Ritalina®, Hipofagin®, Dualid®, Femproporex
**SEDATIVOS BARBITÚRICOS**	Gardenal®, Hidantal®, fenobarbital
**ESTEROIDES ANABOLIZANTES**	Winstrol®, Androxon®, Nebido®, Durateston®, Estandron®, Deca-durabolim®, Deposteron®, Testex®
**ANALGÉSICOS OPIÁCEOS**	Tylex®, Dolantina®, Codein®, Codex®
**ANTICOLINÉRGICOS**	Artane®, Akineton®, Atropina®
**QUETAMINA**	Dopalen®

LISTA Nº2: DROGAS (QUE NÃO ÁLCOOL)

**CANNABIS**	Maconha, Haxixe, Óleo Skank
**ANFETAMINA**	Remédio para ficar acordado (rebite)
**QUETAMINA**	Special K, Super K
**CHÁS**	Ayahuasca, Santo Daime
**COCAÍNA**	Crack Pó, Merla, Oxi, Pasta base, Folhas de Coca
**OPIOIDES**	Heroína, Heroína em fumo, Ópio
**ALUCINÓGENOS**	Ecstasy, Bala, LSD, Mescalina, Peiote, PCP, Pó de anjo, Cristal, DMT
**SOLVENTES OU INALANTES**	Lança-perfume, Loló, Cola de sapateiro, Acetona, Thinner, Éter, Fluido de isqueiro
**GHB**	GHB, Boa noite cinderela, Droga do Estupro, Poppers (Nitrato de Amila), Gás do riso (Halotano)

Listas baseadas em:

Fundação Oswaldo Cruz - Instituto de Comunicação e Informação Científica e Tecnológica em Saúde. *III Levantamento Nacional sobre o uso de drogas pela população brasileira*. 2017. Disponível em <https://www.arca.fiocruz.br/handle/icict/34614>.
